# High‐Performance Daytime Radiative Cooler and Near‐Ideal Selective Emitter Enabled by Transparent Sapphire Substrate

**DOI:** 10.1002/advs.202001577

**Published:** 2020-08-18

**Authors:** Dongwoo Chae, Soomin Son, Yuting Liu, Hangyu Lim, Heon Lee

**Affiliations:** ^1^ Department of Materials Science and Engineering Korea University Anam‐ro 145, Seonguk‐gu Seoul 136‐713 Republic of Korea

**Keywords:** atmospheric transparency window, daytime radiative cooling, selective emitters, transparent sapphire substrates

## Abstract

Daytime radiative cooling serving as a method to pump heat from objects on Earth to cold outer space is an attractive cooling option that does not require any energy input. Among radiative cooler structures, the multilayer‐ or photonic‐structured radiative cooler, composed of inorganic materials, remains one of the most complicated structures to fabricate. In this study, transparent sapphire‐substrate‐based radiative coolers comprising a simple structure and selective emitter‐like optical characteristics are proposed. Utilizing the intrinsic optical properties of the sapphire substrate and adopting additional IR emissive layers, such as those composed of silicon nitride thin film or aluminum oxide nanoparticles, high‐performance radiative coolers can be fabricated with a low mean absorptivity (3–4%) at 0.3–2.5 µm and a high mean emissivity of over 90% at 8–13 µm. Experiments show that the fabricated radiative coolers reach temperature drops of ≈10 °C in the daytime. From the theoretical calculations of radiative cooling performance, the sapphire‐substrate‐based radiative coolers demonstrate a net cooling power as high as 100 Wm^−2^.

## Graphical abstract

Sapphire substrate‐based radiative coolers are introduced having simple structure and selective emitter‐like optical characteristics. Utilizing the intrinsic optical properties of sapphire substrate and additional IR emissive materials, high‐performance radiative coolers composed of purely inorganic materials are presented without elaborate simulation work previous studies reported.

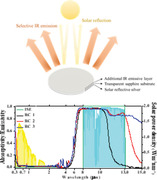

## 1 Introduction

With the advancement in industrialization and acceleration of global warming, the demand for energy required for temperature control is rapidly increasing, thereby reflecting the importance of temperature control. Conventional cooling systems, such as air‐conditioners consume enormous amounts of energy based on fossil fuels, which, in turn, is a shortcut to accelerating global warming by greenhouse gas emission.^[^ ^1^, ^2^, ^3^, ^4^ ^]^

Unlike the aforementioned cooling system, passive radiative cooling technology that harnesses the optical properties of materials for cooling has recently been spotlighted, as it does not require any energy input.^[^ ^5^, ^6^, ^7^ ^]^ Sunlight, with a wavelength range of 0.3–2.5 µm, serves as a heat source in the daytime; meanwhile, the atmospheric transparency window of wavelengths (8–13 µm) is an allowed pathway through which a passive radiative cooler can cool by emitting thermal radiation into cold outer space (*T* = 3 K).^[^ ^8^, ^9^ ^]^ Thus, high‐performance radiative coolers should have high reflectance in the solar spectral region (0.3–2.5 µm) and high emissivity, the same as absorptivity according to Kirchhoff's law of thermal radiation, in the atmospheric transparency window (8–13 µm).^[^ ^10^ ^]^ Extensive studies have been conducted on radiative coolers using thin‐layer deposited films, photonic crystals, metamaterials, nanoparticles (NPs), and polymer‐based composites.^[^ ^4^, ^5^, ^8^, ^9^, ^11^, ^12^, ^13^, ^14^, ^15^, ^16^, ^17^, ^18^, ^19^, ^20^, ^21^, ^22^, ^23^, ^24^, ^25^, ^26^, ^27^, ^28^, ^29^, ^30^ ^]^ In 2014, daytime radiative cooling was spotlighted when Fan et al. reported a photonic radiative cooler that had a sophisticatedly engineered structure with alternatively stacked dielectric layers on a silver‐deposited silicon substrate.^[^ ^5^ ^]^ The photonic radiative cooler exhibited low absorptivity (3%) in the solar spectral region and high emissivity in the atmospheric transparency window, enabling subambient daytime radiative cooling with temperature drops of 4.9 °C and an experimentally determined cooling power of 40.1 Wm^−2^ under direct sunlight. This study led to daytime radiative cooling research that ranged from theoretical design to experimental demonstration. Yin et al. reported a glass‐polymer hybrid metamaterial by utilizing the strong Fröhlich resonance of silica microspheres for daytime radiative cooling.^[^ ^23^ ^]^ The metamaterial radiative cooler provided a powerful experimental cooling power of 93 Wm^−2^ during daytime with a highly facile fabrication process of roll‐to‐roll printing, which enabled the realization of large‐scale demonstration. Subsequently, many types of materials and systems with simple structures have been proposed for fabrication of polymer‐based radiative coolers with broadband emitter‐like optical characteristics and adopted to real‐world applications, including those involving the human body and water cooling.^[^ ^4^, ^8^, ^25^, ^26^, ^27^, ^28^, ^29^ ^]^ Meanwhile, photonic crystal‐structured radiative coolers, mainly composed of inorganic materials in the form of thin‐layer deposited film, still remain as complex structures that require rigorously determined thicknesses for each layer to achieve high emissivity in the atmospheric transparency window and, as such, are difficult to fabricate.^[^ ^14^, ^18^, ^30^ ^]^ YANG, Yang et al. suggested bulk lithium fluoride substrate with backside silver reflective layer which exhibits good optical properties for daytime radiative cooling to address this complicity of radiative cooling structure composed of inorganic materials.^[^ ^21^ ^]^

Selective emitter‐like optical characteristics can be observed in sample structures from previous studies of thin‐layer deposited films, photonic crystals, and bulk material;^[^ ^5^, ^11^, ^12^, ^13^, ^14^, ^15^, ^16^, ^17^, ^18^, ^19^, ^30^ ^]^ meanwhile, broadband emitter‐like optical characteristics with high emissivity in the IR region (2.5–15 µm), which contains the atmospheric transparency window, can be identified in polymer‐based radiative cooling studies.^[^ ^8^, ^23^, ^24^, ^25^, ^26^, ^27^, ^28^, ^29^ ^]^ Selective emitters have been found to be superior in cooling compared with broadband emitters, as they are hardly affected by atmospheric emission.^[^ ^30^ ^]^ Furthermore, considering that selective emitters are mainly composed of inorganic materials with intrinsically good mechanical properties and absence of peeling or yellowing issues, selective emitters have a long‐life span compared with polymer‐based broadband emitters for passive radiative cooling.

Herein, we proposed a simple‐structured and spectrally selective emitter using a transparent sapphire substrate for daytime radiative cooling. The few‐hundred‐µm‐thick sapphire substrate has spectrally selective optical characteristics, namely, low absorptivity in the solar spectral region due to the high bandgap energy of aluminum oxide (≈9 eV) and high emissivity in part of the atmospheric transparency window (8–10 µm). By adopting an additional IR emissive layer with materials that intrinsically have high emissivity in the residual parts of the atmospheric transparency window (10–13 µm) and low absorptivity in the solar spectral region, such as silicon nitride (Si_3_N_4_) or aluminum oxide (Al_2_O_3_), a near‐ideal or high‐performance selective emitter can be easily fabricated. Although the substrate of the selective emitter should be fixed to transparent sapphire substrate unlike the previous studies of multilayer‐structured radiative coolers where various substrate can be used, the fabricated selective emitter exhibits superior optical properties, providing emissivity greater than 90% in the atmospheric transparency window while exhibiting extremely low absorptivity (3–4%) in the solar spectral region.^[^ ^5^, ^13^, ^16^, ^17^, ^18^, ^19^ ^]^ The proposed radiative coolers were experimentally applied to daytime radiative cooling and demonstrated temperature drops of ≈10 °C under direct sunlight. Furthermore, through the theoretical calculation of net cooling power and cooling temperature based on spectral absorptivity and emissivity values, the sapphire‐substrate‐based radiative coolers were demonstrated to be highly efficient with strong reflection of sunlight and emission of thermal radiation, approaching a net cooling power of 100 Wm^−2^.

## 2 Results and Discussion

**Figure 1:**
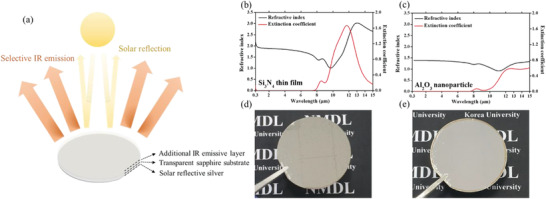
a) Schematic image of sapphire‐substrate‐based radiative cooler with structure of an additional IR emissive layer on the sapphire substrate. Measured refractive index and extinction coefficient value versus wavelength graph for b) Si_3_N_4_ thin film and c) Al_2_O_3_ nanoparticle film. Photo of sapphire‐substrate‐based radiative cooler with d) Si_3_N_4_ thin film and e) Al_2_O_3_ nanoparticle film.

We used Si_3_N_4_ thin film and Al_2_O_3_ NPs for the additional IR emissive layers, which are formed on a thin silver layer deposited on the backside of the sapphire substrate to complement the emissivity through the entire atmospheric window. A photograph, X‐ray diffraction (XRD) 2*θ* scan data, and optical properties such as transmittance, reflectance, absorptance in the solar spectral region and IR region (4–15 µm) of the 430 µm thick transparent sapphire substrate are presented in Figure S1 (Supporting Information). In the photograph, the background image is clearly shown, thereby demonstrating that the sapphire substrate is highly transparent in the visible region (400–700 nm). XRD 2*θ* scan data in Figure S1b (Supporting Information) confirms that the sapphire substrate has *c*‐axis crystallinity, corresponding to a peak at 41.71° with a full width at half maximum of 0.06°. Refractive index and extinction coefficient values of sapphire is shown in Figure S1c (Supporting Information).^[^ ^31^ ^]^ Since the refractive index of sapphire become low after the wavelength of ≈10 µm, the surface of sapphire substrate strongly reflects electromagnetic wave of the wavelength of above 10 µm, acting as a metal. The sapphire substrate has low absorptivity in the entire solar spectral region. In Figure S1e (Supporting Information), the absorptance is shown to be very high, with values near one at 8–10 µm; the value decreases rapidly as the wavelength increases. The transmittance in the wavelength band of 4–8 µm can be converted to reflectance by the deposition of a thin silver layer underneath the sapphire substrate, which eventually provides selective‐emitter‐like optical characteristics to the sapphire‐substrate‐based radiative cooler. The refractive index and extinction coefficient versus wavelength graphs of the adopted additional IR emissive layer (Si_3_N_4_ thin film and Al_2_O_3_ NPs) are presented in **Figure** **1**b,c. The extinction coefficient is proportional to the absorptivity, which means that a low extinction coefficient in the solar spectral region and high extinction coefficient in the atmospheric transparency window is beneficial to daytime radiative cooling. The extinction coefficient values of Si_3_N_4_ thin film are extremely low in the solar spectral region away from the wavelength region around 0.3 µm and broadly high in most of the atmospheric transparency window; this demonstrates that the Si_3_N_4_ thin film has significant potential as a material for radiative cooling, as reported.^[^ ^32^ ^]^ Al_2_O_3_ NPs have a low refractive index and low extinction coefficient values compared with the Si_3_N_4_ thin film, as the Al_2_O_3_ is in the form of an NP. The extinction coefficient of Al_2_O_3_ NP exhibits a small peak at the wavelength of 8.5 µm and increases above the wavelength of 10 µm. In Figure 1d,e, the photographs show the clear mirror‐like color of the sapphire‐substrate‐based radiative cooler with Si_3_N_4_ thin film and Al_2_O_3_ NP. Since the refractive index values of Al_2_O_3_ NP in Figure 1c at the wavelength band of above 10 µm is much higher than that of sapphire in Figure S1c (Supporting Information), the surface of Al_2_O_3_ NP does not reflect the light at the wavelength band, thereby the electromagnetic is eventually absorbed by Al_2_O_3_ NP having high extinction coefficient.

Three sapphire‐substrate‐based radiative coolers were fabricated by plasma‐enhanced chemical vapor deposition for the Si_3_N_4_ layer and spin coating of Al_2_O_3_ NP dispersion for the Al_2_O_3_ NP layer. Sample structures and denoted names are presented in **Table** **1**. The fabrication processes are explained in the Experimental Section below. Because the extinction coefficient for the Al_2_O_3_ NP layer is low compared with that for the Si_3_N_4_ thin film, the Al_2_O_3_ NP layer is fabricated to be thicker. Cross‐sectional Scanning Electron Microscopy (SEM) images of sapphire‐substrate‐based radiative coolers (RC 2 and RC 3) and their silver layers on the backside are shown in Figure S2 (Supporting Information). Without the deposition of platinum layers for SEM image capture, the Si_3_N_4_ thin film and Al_2_O_3_ NP layers are confirmed to have thicknesses of 200 and 1400 nm, respectively. In Figure S3 (Supporting Information), energy dispersive X‐ray spectroscopy (EDS) mapping images of RC 2 and RC 3 are presented with the original captured SEM images. The Si_3_N_4_ thin film is clearly characterized by elemental mapping with atoms of silicon and nitrogen in Figure S3bc (Supporting Information). In Figure S3e,f (Supporting Information), aluminum and oxygen are shown in the entire captured image, which covers the sapphire substrate.

**Table 1:** Denoted name and structure configuration of RC 1, RC 2, and RC 3

Denoted name	RC 1	RC 2	RC 3
Structure configuration	Transparent sapphire substrate (430 µm) with backside silver layer (200 nm)	Si_3_N_4_ (200 nm) thin film on transparent sapphire substrate (430 µm) with backside silver layer (200 nm)	Al_2_O_3_ NP (R_Al2O3_ = 10–20 nm) layer (1400 nm) on transparent sapphire substrate (430 µm) with backside silver layer (200 nm)

**Figure 2:**
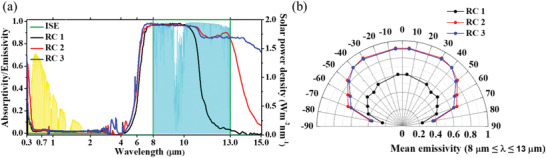
a) Absorptivity/emissivity spectra of ISE, RC 1, RC 2, and RC 3. The backside yellow‐ and cyan‐colored spectra are the solar power density of AM 1.5 Direct + Circumsolar condition and atmospheric transmittance in the atmospheric transparency window, which are in Figure S5 (Supporting Information), respectively. b) Mean emissivity in atmospheric transparency window according to incident angle graph for RC 1, RC 2, and RC 3.

The absorptivity/emissivity spectra of the ideal selective emitter (ISE) and sapphire‐substrate‐based radiative coolers (RC 1, RC 2, and RC 3), and the averaged optical properties for RC 1, RC 2, and RC 3 are presented in **Figure** **2**a and **Table** **2**, respectively. The solar power density and atmospheric transmittance graphs, which are each colored with yellow and cyan in the background, respectively, are shown in Figure S5 (Supporting Information).^[^ ^33^ ^]^ ISE has an absorptivity of zero in the solar spectral region and an emissivity of one in the atmospheric transparency window, meaning that it is not affected by sunlight as a heat source. RC 1, which is the 430 µm thick sapphire substrate with a 200 nm thick silver layer on the backside, has an extremely low absorptivity of 2.8% in the solar spectral region and high emissivity in part of the atmospheric transparency window (8–10 µm). With the deposition of the silver layer, the transmittance at 4–8 µm of the sapphire substrate is converted to reflectance, as shown in Figure S1c (Supporting Information). The absorptivity/emissivity spectra and averaged optical properties for the 220 µm thick sapphire substrate is shown in Figure S4 and Table S1 (Supporting Information). The RC 1 structure with 220 µm thick sapphire is better for achieving the selective‐emitter‐like optical characteristics than the 430 µm thick sapphire substrate. However, the 220 µm thick sapphire substrate is much more expensive, owing to additional steps in the production process, such as chemical‐mechanical polishing. Therefore, we use 430 µm thick sapphire substrate instead for the radiative cooling samples. By adding the Si_3_N_4_ thin film and Al_2_O_3_ NP layer to the sapphire substrate, corresponding to RC 2 and RC 3, the emissivity in the wavelength range of 10–13 µm provides more favorable optical properties for daytime radiative cooling. Both radiative coolers have strong emission properties in the atmospheric transparency window while maintaining highly reflective behaviors for the wavelength band of the solar spectral region. For RC 2, the thickness of the Si_3_N_4_ thin film is determined in accordance to Figure S6 (Supporting Information), which shows that the RC 2 structure with 200 nm thick Si_3_N_4_ thin film exhibits optimal radiative cooling performance with balanced absorptivity and emissivity in each targeted region. The processed absorptivity and emissivity in Table 2 are 4.3% and 91.6% for RC 2 and 3.5% and 90.8% for RC 3, respectively. The spectra in the IR region shows that RC 2 exhibits a closer fit to the ISE than RC 3, since the extinction coefficient values of the Si_3_N_4_ thin film become low beyond the wavelength of 13 µm, which is different from that of the Al_2_O_3_ NP layer, as shown in Figure 1c,d (Supporting Information). However, in the solar spectral region, RC 2 exhibits higher absorptivity than RC 3 because of the low bandgap energy of Si_3_N_4_ (≈5 eV) compared with that of Al_2_O_3_ (≈9 eV).^[^ ^34^, ^35^ ^]^ The mean emissivity in atmospheric transmittance versus incident angle graph is presented in Figure 2b. RC 2 has a more angle‐robust emissive property than RC 3 for incident angles over 50°. However, both radiative coolers show emissivities as high as 0.6 up to an incident angle of 70°.

**Table 2:** Averaged mean absorptivity in solar spectral region and mean emissivity in atmospheric transparency window of RC 1, RC 2, and RC 3

	RC 1	RC 2	RC 3
*α*_mean_(0.3 − 2.5 µm)	0.028	0.043	0.035
*ɛ*_mean_(8 − 13 µm)	0.610	0.916	0.908

**Figure 3:**
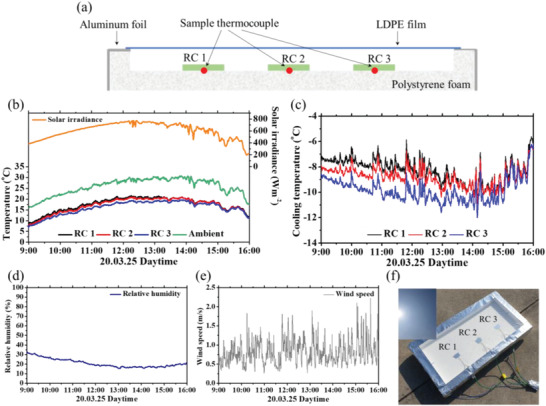
a) Schematic image of external measurement chamber. b) Temperature profile of RC 1, RC 2, RC 3, and ambient, c) measured cooling temperature (*T*_cool_) calculated with the equation of *T*_cool_ = *T*_sample_ − *T*_ambient_, d) relative humidity, e) wind speed data logged in daytime from 9:00 to 16:00. f) Photograph in measured time; the inset image shows the sky is clear.

The outdoor temperature was measured on a rooftop of a building at the Korea University campus (37° 34′ 56.8” N, 127° 01′ 35.3” E). **Figure** **3** shows a schematic of the temperature measurement apparatus, logged temperature profile, solar irradiance, cooling temperature, relative humidity, and wind speed, and a photograph of the temperature measurement apparatus with an inset image of a clear sky. Before RC 1, RC 2, and RC 3 were applied to the outdoor temperature measurement, their edge sides were sliced in order that the samples would be parted in the center, as the edge sides were tinged with a certain color due to the fabrication process (e.g., sputtering, spin‐coating). The body of the measurement apparatus is composed of white compressed polystyrene with a thickness of 30 mm, and its outer side is covered with aluminum tape to prevent solar heating. K‐type adhesive thermocouples (ST‐50, RKC INSTRUMENT INC., Japan) are attached to the backside each RC and placed on the polystyrene body. A copper wire ambient thermocouple was placed at the edge side of the apparatus to measure the real in‐apparatus temperature. Because the low‐density polyethylene (LDPE) film is covered on the top of the apparatus, the greenhouse effect, which makes the temperature inside the apparatus hot, occurs due to nonideal transmittance of the LDPE film, shown in Figure S5c (Supporting Information). The temperature of RC 1, RC 2, RC 3 and ambient were logged every 30 s with a data logger (OM‐CP‐OCTTEMP‐A, OMEGA Engineering, USA). In addition, solar irradiance, relative humidity, and wind speed were logged every 30 s with a weather station (HD52.3D, DeltaOHM, Italy). The logged temperature profile of RC 1, RC 2, RC 3, and ambient and the solar irradiance in the daytime from 09:00 to 16:00 are presented in Figure 3b. The calculated cooling temperatures, shown as negative values, the relative humidity, and wind speed during the day are presented in Figure 3c–e, respectively. The weather was clear, as shown in the inset image of Figure 3f. During that day, all radiative coolers exhibited subambient radiative cooling effects compared with the ambient temperature. The average temperature drops of RC 1, RC 2, and RC 3 were −8.24, −8.75, and −9.82 °C, respectively. RC 3 showed the most powerful cooling effect among the three radiative coolers with a conformity of absorptivity/emissivity spectra results, which indicate that RC 3 has lower absorptivity in the solar spectral region than RC 2 and higher emissivity in the atmospheric transparency window than RC 1. Although RC 2 exhibits strong emission properties in the atmospheric transparency window, the cooling performance of RC 2 was not much different from that of RC 1 (≈0.5 °C) owing to the high solar absorption induced by the Si_3_N_4_ thin film. For RC 1, daytime radiative cooling could be achieved with the deposition of a silver layer on the backside of the sapphire substrate.

**Figure 4:**
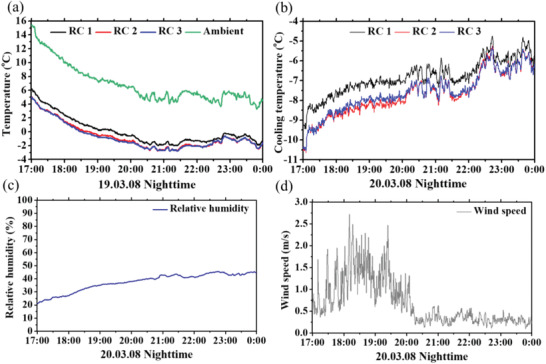
a) Temperature profile of RC 1, RC 2, RC 3, and ambient, b) calculated cooling temperature, c) relative humidity, d) wind speed logged in nighttime from 17:00 to 00:00.

The same measurement was conducted in the nighttime from 17:00 to 00:00, as presented in **Figure** **4**. During nighttime, the measured solar irradiance values were near‐zero or zero, and the relative humidity was higher than that in the daytime. At 17:00 when the sun set below the horizon, the radiative cooling performances for each radiative cooler were the highest due to the low solar irradiance and relative humidity and the high ambient temperature. RC 1, which has the lowest emissivity in the atmospheric transparency window among the three radiative coolers, exhibited the lowest radiative cooling performance compared with RC 2 and RC 3 by ≈1.5 °C at 17:00. RC 1, RC 2, and RC 3 had averaged temperature drops of −6.81, −7.67, and −7.57 °C, respectively, and RC 2 exhibited the highest temperature drops over the entire measurement period. Since the selective emitter exhibits superior cooling temperature performance compared with the broadband emitter, RC 2 with more selective‐emitter‐like optical characteristics compared with RC 3 exhibited higher temperature drops.^[^ ^36^ ^]^ As time passed, the cooling temperature differences between RC 1, RC 2, and RC 3 decreased. The increasing relative humidity and decreasing ambient temperature caused the degradation of radiative cooling performance by reducing the atmospheric transmittance and thermal radiation power, respectively.

**Figure 5:**
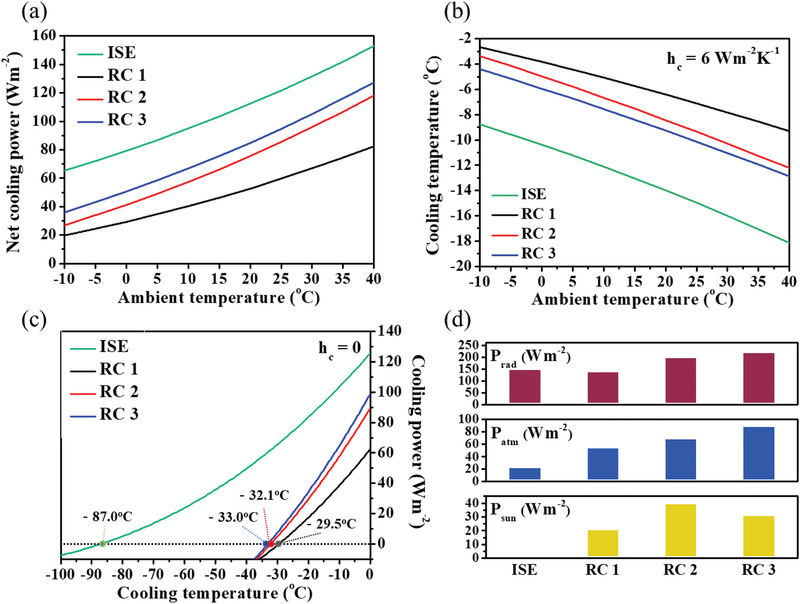
a) Net cooling power and b) cooling temperature versus ambient temperature graphs of ISE, RC 1, RC 2, and RC 3 for daytime. c) Cooling temperature versus cooling power graph under conditions of zero heat transfer coefficient and ambient temperature of 300 K. d) Radiation power, atmospheric emission power, and solar absorption power values of ISE, RC 1, RC 2, and RC 3 for daytime.

**Figure** **5**a,b presents the calculated net cooling power (*P*_net_) and cooling temperature in daytime. The calculated values of calculated cooling power and cooling temperature in Figure 5; and Figures S6 and S7 (Supporting Information) were produced from the atmospheric transmittance values in Figure S5b (Supporting Information) with the wavelength range from 0.3 to 15 µm. Heat transfer coefficient of 6 Wm^−2^ K^−1^ was selected based on previous works for clear day with wind speed around 1 m s^−1^.^[^ ^5^, ^17^, ^37^, ^38^ ^]^ At an ambient temperature of 27 °C (300 K), the calculated net cooling power of ISE, RC 1, RC 2, and RC 3 were 125.7, 62.5, 89.5, and 98.9 Wm^−2^, respectively. Since radiation power is proportional to the surface temperature, the net cooling power increases with ambient temperature. It should be noted that even the cooling power of ISE does not approach 130 Wm^−2^. Although RC 1 shows the lowest slope, in accordance with the relatively low spectral emissivity in the atmospheric transparency window, RC 1, which has a simple structure with a silver‐deposited sapphire substrate, exhibits a net cooling power as large as 60 Wm^−2^. In addition, RC 3 shows the most remarkable net cooling power of 98.9 Wm^−2^ with lower solar absorption compared with RC 2. In Figure 5b, the calculated cooling temperature of ISE, RC 1, RC 2, and RC 3 are −15.4, −7.39, −9.74, and −10.5 °C, respectively, at the temperature of 300 K. With the same stream for calculated net cooling power, RC 3 exhibits the lowest cooling temperature. The gaps in cooling temperature between the ISE and RC 1, RC 2, and RC 3 are due to the unavoidable intrinsic solar absorption originating from the silver layer and IR emissive layer. The cooling temperature values of the four radiative coolers at an ambient temperature of 300 K and heat transfer coefficient of 0 are shown in Figure 5c. The case of a heat transfer coefficient of zero in Figure 5c can be interpreted as the place near the radiative coolers is in vacuum, and the atmospheric transparency window influences the radiative coolers. An experimental demonstration with a similar concept was proposed by Fan et al.^[^ ^13^ ^]^ In Figure 5c, the cooling temperature values of ISE, RC 1, RC 2, and RC 3 are −87.0, −29.5, −32.1, −33.0 °C, respectively. The power parameters constituting the net cooling power, namely, the radiation power (*P*_rad_), atmospheric emission power (*P*_atm_), and solar absorption power (*P*_sun_) are presented in Figure 5d and **Table** **3**.

**Table 3:** Radiation power, atmospheric emission power, solar absorption power, and net cooling power of ISE, RC 1, RC 2, and RC 3 with an ambient temperature (*T*_amb_) of 300 K

*T*_amb_ = 300 K	ISE	RC 1	RC 2	RC 3
*P*_rad_(Wm^−2^)	148.1	137.2	197.6	217.6
*P*_atm_(Wm^−2^)	22.5	54.1	68.7	88.1
*P*_sun_(Wm^−2^)	0	20.6	39.5	30.7
*P*_net_(Wm^−2^)	125.7	62.5	89.5	98.9

RC 3 can exhibit theoretical temperature drops of −33 °C in the daytime with remarkable radiative cooling properties. The radiation power of ISE is not the highest value among the radiative coolers. However, the radiation power of ISE, which originates from the entire atmospheric transparency window, is solely used to cool the surface of the ISE, indicating that the cooling temperature of ISE is near −90 °C. The net cooling power, cooling temperature, and cooling temperature versus cooling power graphs at nighttime are shown in Figure S7 (Supporting Information). At night time with no effect of solar irradiance, RC 2 is more effective in radiative cooling than RC 3, with a low atmospheric emission power demonstrated by the spectral fit to ISE. In addition, RC 2 exhibits a cooling temperature of −52.7 °C for a heat transfer coefficient of zero. Considering the theoretical calculation, for radiative cooling, RC 2 and RC 3 demonstrate superiority at nighttime and daytime, respectively. To investigate the effect of atmospheric transmittance with different season or location characterized by some factors such as ground temperature, water vapor column, and altitude, the six different atmospheric transmittance model are provide by MODTRAN 6 software in Figure S8 (Supporting Information).^[^ ^39^ ^]^ The calculated net cooling power and cooling temperature graphs of ISE, RC 1, RC 2, RC 3 for those different atmospheric transmittance models are presented in Figure S9 (Supporting Information), showing significant difference in results by location and season. Several factors, such as sample structure, radiative cooling performance parameters (*α*_mean_(0.3 − 2.5 µm), *ɛ*_mean_(8 − 13 µm), *P*_net_, *T*_cool_), and measurement conditions for previously reported radiative coolers with the structure of multilayered film, photonic crystal, polymer, and particle embedded polymer matrix and the cooler demonstrated in our work is summarized in **Table** **4**. Without elaborate simulation for a radiative cooler composed of inorganic materials, the proposed radiative cooler using the sapphire substrate as an optical component exhibits superior radiative cooling performance with selective‐emitter‐like optical characteristics and a simple structure that can be completed by just a few deposition processes.

**Table 4:** Summary of previously reported radiative coolers and our work for sample structure, radiative cooling performance parameters, and measurement condition

Reference	Structural configuration (From top to bottom)	Method	*α*_mean_ [0.3–2.5 µm]	*ε*_mean_ [8–13 µm]	*P*_net_ [Wm^−2^]	*T*_cool_ [°C]	Measurement location	Measurement condition
^[^ ^5^ ^]^	SiO_2_ (230 nm), HfO_2_ (485 nm), SiO_2_ (688 nm), HfO_2_ (13 nm), SiO_2_ (73 nm), HfO_2_ (34 nm), SiO_2_ (54 nm) and Ag (200 nm) on Si substrate	Theoretical and experimental	0.03	0.68	40.1	4.9	California, USA	Nonvacuum
^[^ ^8^ ^]^	Porous poly(vinylidene fluoride‐*co*‐hexafluoropropene) (⪰ 300 µm)	Experimental	0.04	0.97	96	6	Phoenix, USA	Nonvacuum
^[^ ^11^ ^]^	*α*‐Quartz (2.5 µm) and SiC (8 µm) with periodicity of 6 µm and width mesh patterns of 5.4 µm, and 3 sets of 5 bilayers with TiO_2_ and MgF_2_ on Ag	Theoretical	0.035	N.A	105	N.A	N.A	N.A
^[^ ^13^ ^]^	Si_3_N_4_ (70 nm), Si (700 nm), and Al (150 nm) on Si substrate	Experimental	N.A	0.85	54	42	California, USA	Vacuum (ZnSe window) and Al solar shade
^[^ ^14^ ^]^	TiO_2_ (200 nm) and SiO_2_ (200 nm) four pairs on Ag (200 nm)	Theoretical	0.008	0.84	85.5	N.A	N.A	N.A
^[^ ^16^ ^]^	SiO_2_ (500 nm) and TiO_2_ (500 nm) four pairs and Ag (200 nm) on Si substrate	Theoretical and experimental	0.06	0.84	96.4	7.2	Hong Kong	Nonvacuum and Al solar shade
^[^ ^17^ ^]^	SiO_2_ (276 nm), Si_3_N_4_ (312 nm), Al_2_O_3_ (1312 nm), and Ag (200 nm) on Si substrate	Theoretical and experimental	0.05	0.87	66.0	6.2	Seoul, South Korea	Nonvacuum
^[^ ^18^ ^]^	SiO_2_ (314 nm), TiO_2_ (782 nm), SiO_2_ (901 nm), TiO_2_ (797 nm), SiO_2_ (880 nm), TiO_2_ (378 nm), SiO_2_ (951 nm) and Ag (100 nm) on Si substrate	Theoretical	0.03	0.80	100	N.A	N.A	N.A
^[^ ^23^ ^]^	SiO_2_ microsphere embedded polymethyl‐pentene (50 µm) and Ag (200 nm)	Theoretical and experimental	0.04	0.93	93	N.A	Arizona, USA	Nonvacuum
^[^ ^28^ ^]^	Epoxy resin (50 µm) and Ag (300 nm) on SiO_2_ substrate	Experimental	0.07	0.92	33	2.7	Tianjin, China	Nonvacuum, humid weather
This work	Al_2_O_3_ NP layer (1400 nm), Sapphire substrate (430 µm), and Ag (200 nm)	Experimental	0.035	0.91	98.9	9.8	Seoul, South Korea	Nonvacuum

## 3 Conclusion

In summary, we proposed simple‐structured transparent sapphire‐substrate‐based radiative coolers using the sapphire substrate as an optical component. With intrinsic optical properties of the sapphire substrate, such as low absorptivity and high emissivity in part of the atmospheric transparency window and additional IR emissive layers composed of Si_3_N_4_ or Al_2_O_3_ nanoparticle to reinforce IR emission, high‐performance radiative cooler and near‐ideal selective emitter can be easily fabricated with a few deposition process. The sapphire substrate‐based radiative coolers exhibit emissivity of over 90% in the entire atmospheric transparency window and absorptivity as low as 4% in the solar spectral region, as well as angle‐robust emissive properties. In daytime under clear sky conditions, the sapphire‐substrate‐based radiative cooler showed temperature drops of ≈10 °C compared with ambient temperature. In addition, the calculated net cooling power value was nearly 100 Wm^−2^, demonstrating the great potential of the simple‐structured sapphire‐substrate‐based radiative cooler for daytime radiative cooling.

## 4 Experimental Section

##### Sample Preparation

Transparent and double‐side‐polished sapphire substrate with thicknesses of 220 and 430 µm were acquired from Hi Solar in South Korea. Si_3_N_4_ thin film was deposited by plasma‐enhanced chemical vapor deposition (Plasmalab800Plus, Oxford Instrument, UK) at a temperature of 250 °C and a deposition speed of 0.5 nm s^−1^. Al_2_O_3_ NPs had a mean particle size in the range of 10–20 nm and were used in the form of a dispersion solution with ethanol as the solvent at a weight ratio of 20%. The Al_2_O_3_ NP layer was spin‐coated on the sapphire substrate with a rotation speed of 3000 rpm and rotation time of 30 s (VSF‐200MD, RHABDOS, South Korea) and soft‐baked at 80 °C for 5 min to evaporate the remaining ethanol solvent. After the deposition of the IR emissive layers, a 200 nm thick solar reflective silver layer was deposited on the backside of the transparent sapphire substrate by sputtering (KVS‐T406038 Magnetron, KOREA VACUUM TECH, South Korea). Subsequently, a 20 nm thick aluminum layer was formed by sputtering for the protection of the silver layer and adhesion between the deposited layers and sapphire substrate.

##### Sample Characterization

An XRD 2*θ* scan (SmartLab, Rigaku, Japan) was conducted using an X‐ray diffractometer with a Cu K_*α*_ X‐ray source, which had a wavelength of 1.5412 Å and X‐ray power of 9 kW (45 kV/200 mA). Optical properties in the solar spectral region were measured by a UV–Vis–NIR spectrophotometer (Solidspec‐3700, Shimadzu, Japan) with an integrating sphere, and a protected silver mirror (ME2S‐P01, Thorlabs, USA) was used as a baseline reflector. After measuring the reflectance (R) and transmittance (T), the measured reflectance and transmittance was converted to absolute reflectance and transmittance by multiplication of the absolute reflectance of the protected silver mirror. Finally, absorptance (A) was calculated using the equation: *A* = 1 − *R* − *T*. The optical properties in the IR region were measured by Fourier transform infrared spectroscopy (FT‐IR) (Nicolet IS‐50, Thermo Scientific, USA) with an integrating sphere (Mid‐IR IntegratIR, PIKE Technologies, USA), and a standard gold reflector was used for the baseline measurement. For optical properties according to the incident angle in the IR region, an FT‐IR accessory (VeeMAX III, PIKE Technologies, USA) was used. The absorptance in the IR region was acquired by the above equation. Because the transmittance of the 200 nm thick silver layer in the wavelength band from 0.3 to 15 µm is nearly zero, transmittance measurements were not conducted for samples with a thin silver layer backside, and the absorptivity of those was calculated by the equation: *A* = 1 − *R*. Refractive index and extinction coefficient values of Si_3_N_4_ thin film and Al_2_O_3_ NPs were obtained through spectroscopic ellipsometry (IR‐VASE Mark II Ellipsometer, J.A. Woollam, USA). 100 nm thick Si_3_N_4_ thin film and Al_2_O_3_ NPs on silicon substrate were used to measurement. Cross‐sectional SEM images were acquired by SEM instrument (Hitachi 4800, Hitachi, Japan) with a vacuum voltage of 5 kV and current of 15 mA, and EDS elemental mapping images were acquired using the instrument EMAX (Horiba, England) with an X‐ray source of Cu K_*α*_.

##### Radiative Cooling Performance Evaluation

The radiative cooling equation can be expressed by the following equation, which is based on the law of conservation of energy^[^ ^5^, ^6^, ^7^, ^36^ ^]^

(1) $$\begin{array}{c} P_{\mathrm{net}}\left(T\right)=\;P_{\mathrm{rad}}\left(T\right)-P_{\mathrm{atm}}\left(T_{\mathrm{atm}}\right)-P_{\mathrm{sun}}-P_{\mathrm{non}-\mathrm{rad}} \end{array}$$

where

(2) $$\begin{array}{c} P_{\mathrm{rad}}\left(T\right)=\;\int_{0}^{2\pi }\int_{0}^{\frac{\pi }{2}}\int_{0}^{\infty }I_{\mathrm{BB}}\left(T,\lambda \right)\varepsilon \left(\lambda ,\theta \right)\cos\theta \sin\theta \;d\lambda d\theta d\varphi \end{array}$$

is the hemispheric radiation power emitted from the surface of a radiative cooler.

(3) $$\begin{array}{ccc} & & P_{\mathrm{atm}}\left(T_{\mathrm{atm}}\right)\;\\ & & =\int_{0}^{2\pi }\int_{0}^{\frac{\pi }{2}}\int_{0}^{\infty }I_{\mathrm{BB}}\left(T_{\mathrm{atm}},\lambda \right)\varepsilon \left(\lambda ,\theta \right)\;\varepsilon_{\mathrm{atm}}\left(\lambda ,\theta \right)\cos\theta \sin\theta d\lambda d\theta d\varphi \; \end{array}$$

is the power loss due to atmospheric radiation absorption of a radiative cooler.

(4) $$\begin{array}{c} P_{\mathrm{sun}}=\int_{0}^{\infty }I_{\mathrm{AM}1.5}\left(\lambda \right)\varepsilon \left(\lambda ,\theta \right)d\lambda \end{array}$$

is the power loss due to solar absorption of a radiative cooler.

(5) $$\begin{array}{c} P_{\mathrm{non}-\mathrm{rad}}=h_{c}\left(T_{\mathrm{atm}}-T\right) \end{array}$$

is the power loss caused by conduction and convection.

Here, blackbody radiation intensity is expressed by the equation: $I_{\mathrm{BB}}=\frac{\frac{2hc^{2}}{\lambda^{5}}}{\left[e^{hc/\lambda k_{B}T}-1\right]}$, and angular atmospheric emissivity is expressed by: $\varepsilon_{\mathrm{atm}}\;\left(\lambda ,\theta \right)=\;1-t{\left(\lambda \right)}^{\frac{1}{\cos(\theta )}}.\;$In these equations the variables of *h*, *c*, *k*_B_, and *h*_c_ represent the Planck constant, speed of light, Boltzmann constant, and heat transfer coefficient, respectively. *ε*(*λ*, *θ*) is the angular emissivity of the film. The cooling temperature was used (Δ*T*_cool_ = *T* − *T*_atm_), which was calculated by the extraction of the cooling temperature under the condition of *P*_net_ (*T*) = 0. *T*_amb_ was used instead of *T*_atm_, as previous studies have reported.^[^ ^29^, ^40^ ^]^ The overall calculation process for the net cooling power and cooling temperature was conducted using MATLAB software based on spectral absorptivity and emissivity values, with a wavelength spacing of 0.01 µm.

## Conflict of Interest

The authors declare no conflict of interest.

## Supporting information

Supporting Information

## References

[1] U.S. Energy Information , Annual Energy Outlook 2015 with Projections to 2040, 2015.

[2] P. Huovila , Buildings and Climate Change: Status, Challenges, and Opportunities, UNEP/Earthprint, London 2007.

[3] E. A. Goldstein , A. P. Raman , S. Fan , Nat. Energy 2017, 2, 17143.

[4] P.‐C. Hsu , A. Y. Song , P. B. Catrysse , C. Liu , Y. Peng , J. Xie , S. Fan , Y. Cui , Science 2016, 353, 1019. 27701110 10.1126/science.aaf5471

[5] A. P. Raman , M. A. Anoma , L. Zhu , E. Rephaeli , S. Fan , Nature 2014, 515, 540. 25428501 10.1038/nature13883

[6] X. Sun , Y. Sun , Z. Zhou , M. A. Alam , P. Bermel , Nanophotonics 2017, 6, 997.

[7] B. Zhao , M. Hu , X. Ao , N. Chen , G. Pei , Appl. Energy 2019, 236, 489.

[8] J. Mandal , Y. Fu , A. C. Overvig , M. Jia , K. Sun , N. N. Shi , H. Zhou , X. Xiao , N. Yu , Y. Yang , Science 2018, 362, 315. 30262632 10.1126/science.aat9513

[9] B. Bhatia , A. Leroy , Y. Shen , L. Zhao , M. Gianello , D. Li , T. Gu , J. Hu , M. Soljacic , E. N. Wang , Nat. Commun. 2018, 9, 5001. 30479326 10.1038/s41467-018-07293-9 PMC6258698

[10] P.‐M. Robitaille , Progr. Phys. 2009, 4, 3.

[11] E. Rephaeli , A. Raman , S. Fan , Nano Lett. 2013, 13, 1457. 23461597 10.1021/nl4004283

[12] M. M. Hossain , B. Jia , M. Gu , Adv. Opt. Mater. 2015, 3, 1047.

[13] Z. Chen , L. Zhu , A. Raman , S. Fan , Nat. Commun. 2016, 7, 13729. 27959339 10.1038/ncomms13729 PMC5159822

[14] M. A. Kecebas , M. P. Menguc , A. Kosar , K. Sendur , J. Quant. Spectrosc. Radiat. Transfer 2017, 198, 179.

[15] G. J. Lee , J. K. Yeong , H. M. Kim , Y. J. Yoo , Y. M. Song , Adv. Opt. Mater. 2018, 6, 1800707.

[16] S. Y. Jeong , C. Y. Tso , J. Ha , Y. M. Wong , C. Y. H. Chao , B. Huang , H. Qiu , Renewable Energy 2020, 146, 44.

[17] D. Chae , M. Kim , P.‐H. Jung , S. Son , J. Seo , Y. Liu , B. J. Lee , H. Lee , ACS Appl. Mater. Interfaces 2020, 12, 8073. 31990166 10.1021/acsami.9b16742

[18] M. A. Kecebas , M. P. Menguc , A. Kosar , K. Sendur , J. Opt. Soc. Am. B 2020, 37, 1173.

[19] H. Ma , K. Yao , S. Dou , M. Xiao , M. Dai , L. Wang , H. Zhao , J. Zhao , Y. Li , Y. Zhan , Sol. Energy Mater. Sol. Cells 2020, 212, 110584.

[20] J.‐L. Kou , Z. Jurado , Z. Chen , S. Fan , A. J. Minnich , ACS Photonics 2017, 4, 626.

[21] Y. Yang , L. Long , S. Meng , N. Denisuk , G. Chen , L. Wang , Y. Zhu , Sol. Energy Mater. Sol. Cells 2020, 211, 110548.

[22] H. Bao , C. Yan , B. Wang , X. Fang , C. Y. Zhao , X. Ruan , Sol. Energy Mater. Sol. Cells 2017, 168, 78.

[23] Y. Zhai , Y. Ma , S. N. David , D. Zhao , R. Lou , G. Tan , R. Yang , X. Yin , Science 2017, 355, 1062. 28183998 10.1126/science.aai7899

[24] T. Li , Y. Zhai , S. He , W. Gan , Z. Wei , M. Heidarinejad , D. Dalgo , R. Mi , X. Zhao , J. Song , J. Dai , Science 2019, 364, 760. 31123132 10.1126/science.aau9101

[25] A. Aili , Z. Y. Wei , Y. Z. Chen , D. L. Zhao , R. G. Yang , X. B. Yin , Mater. Today Phys. 2019, 10, 100127.

[26] D. Fan , S. Hui , L. Qiang , Sol. Energy Mater. Sol. Cells 2019, 195, 250.

[27] S. Meng , L. Long , Z. Wu , N. Denisuk , Y. Yang , L. Wang , F. Cao , Y. Zhu , Sol. Energy Mater. Sol. Cells 2020, 208, 110393.

[28] J. Liu , D. Zhang , S. Jiao , Z. Zhou , Z. Zhang , F. Gao , Sol. Energy Mater. Sol. Cells 2020, 207, 110368.

[29] D. Zhao , A. Aili , Y. Zhai , J. Lu , D. Kidd , G. Tan , X. Yin , R. Yang , Joule 2019, 3, 111.

[30] K. Yao , H. Ma , M. Huang , H. Zhao , J. Zhao , Y. Li , S. Dou , Y. Zhan , ACS Appl. Nano Mater. 2019, 2, 5512.

[31] M. Querry , US Army Chem. Res. Dev. Eng. Cent. (CRDC), Aberdeen Proving Ground, MD 1985.

[32] Z. Liang , H. Shen , J. Li , N. Xu , Sol. Energy 2002, 72, 505.

[33] S. D. Lord , A New Software Tool for Computing Earth's Atmospheric Transmission of Near‐ and Far‐Infrared Radiation, Vol. 103957, Ames Research Center, Moffett Field, CA 1992.

[34] H. S. Nalwa , Silicon‐Based Material and Devices, Two‐Volume Set: Materials and Processing, Properties and Devices, Vol. 1 (Ed: Nalwa H. S.), Academic Press, Cambridge, MA 2001.

[35] W. J. Tropf , M. E. Thomas , Handbook of Optical Constants of Solids, Academic Press, Cambridge, MA 1997, p. 653.

[36] M. M. Hossain , M. Gu , Adv. Sci. 2016, 3, 1500360. 10.1002/advs.201500360 PMC5067572 27812478

[37] T. M. J. Nilsson , G. A. Niklasson , Sol. Energy Mater. Sol. Cells 1995, 37, 93.

[38] J. Liu , J. Zhang , D. Zhang , S. Jiao , J. Xing , H. Tang , Y. Zhang , S. Li , Z. Zhou , J. Zuo , Renewable Sustainable Energy Rev. 2020, 130, 109935.

[39] A. Berk , P. Conforti , R. Kennett , T. Perkins , F. Hawes , J. Van Den Bosch , in 6th Workshop Hyperspectral Image Signal Process. Evol. Remote Sens., Whispers, Lausanne, Switzerland 2014.

[40] A. Aili , D. Zhao , J. Lu , Y. Zhai , X. Yin , G. Tan , R. Yang , Energy Convers. Manage. 2019, 186, 586.

