# Supplementary material for: High‐Performance Daytime Radiative Cooler and Near‐Ideal Selective Emitter Enabled by Transparent Sapphire Substrate
Source: Adv Sci (Weinh). 2020 Aug 18;7(19):2001577. doi: 10.1002/advs.202001577 (PMC7539194; doi:10.1002/advs.202001577)
Supplement: Supplementary file 1 — Supporting Information [file ADVS-7-2001577-s001.pdf]

© 2020 Wiley-VCH GmbH

Supporting Information

for *Adv. Sci.*, DOI: 10.1002/advs.202001577

High-Performance Daytime Radiative Cooler and Near-Ideal Selective Emitter Enabled by Transparent Sapphire Substrate

*Dongwoo Chae, Soomin Son, Yuting Liu, Hangyu Lim, and Heon Lee**

Supporting Information

High-Performance Daytime Radiative Cooler and Near-Ideal Selective Emitter Enabled by Transparent Sapphire Substrate

*Dongwoo Chae, Soomin Son, Yuting Liu, Hangyu Lim, and Heon Lee**

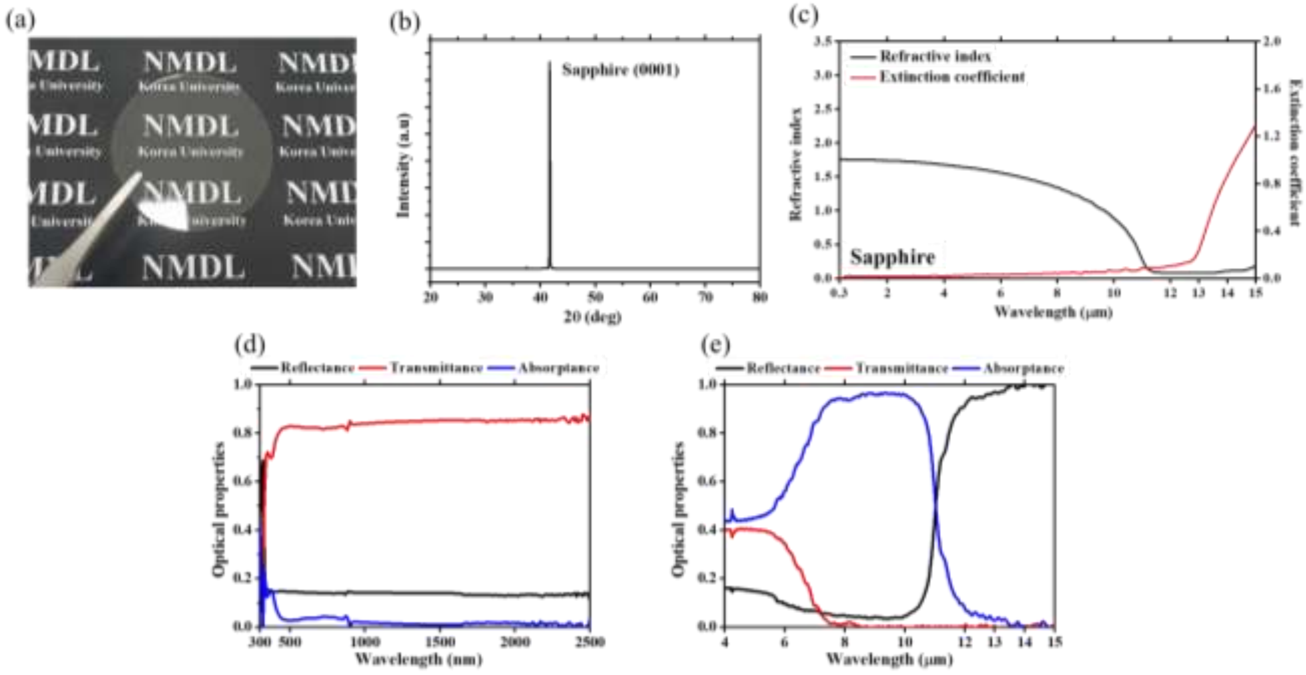

Figure S1. (a) Photograph and (b) XRD data of transparent sapphire substrate. NMDL logo can be clearly seen due to the highly transparent sapphire substrate. (c) Refractive index and extinction coefficient values of sapphire crystal.[1] Optical properties (reflectance, transmittance, and absorptance) in (d) UV-Vis-NIR region (0.3–2.5 μm) and (e) IR region (4–15 μm)

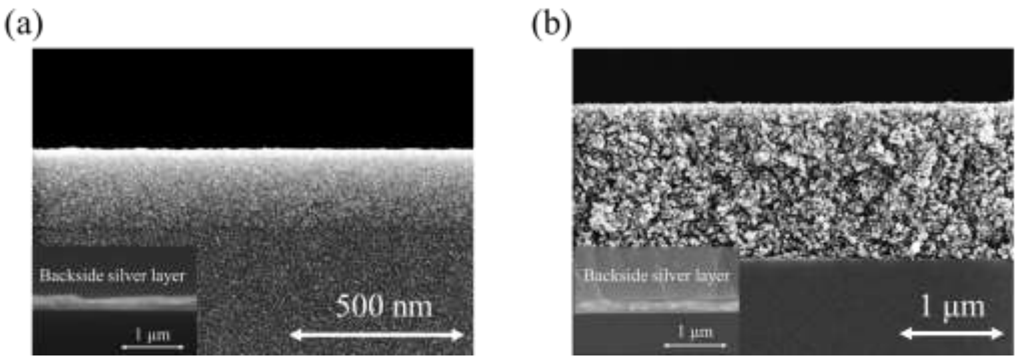

Figure S2. Cross-sectional SEM images of (a) RC 2 and (b) RC 3. The inset images show thin silver layer attached on the backside of the sapphire substrate.

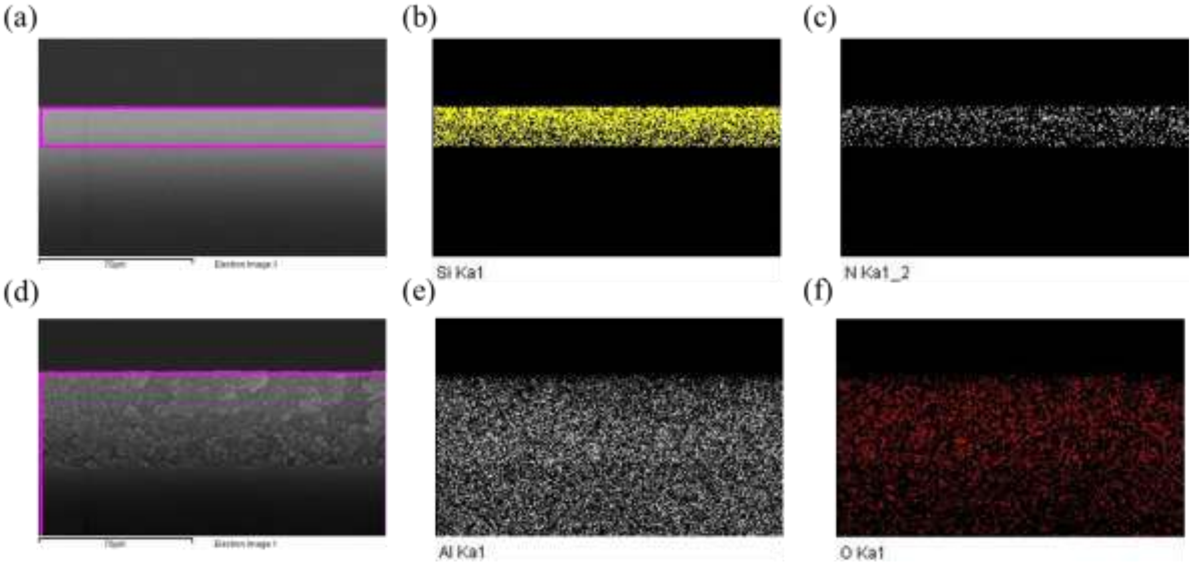

Figure S3. (a) Captured cross-sectional SEM images and EDS elemental mapping image of RC 2 for (b) silicon and (c) nitrogen. (d) Captured cross-sectional SEM images and EDS elemental mapping image of RC 3 for (e) aluminum and (f) oxygen. The sapphire substrate is confirmed by elemental mapping images.

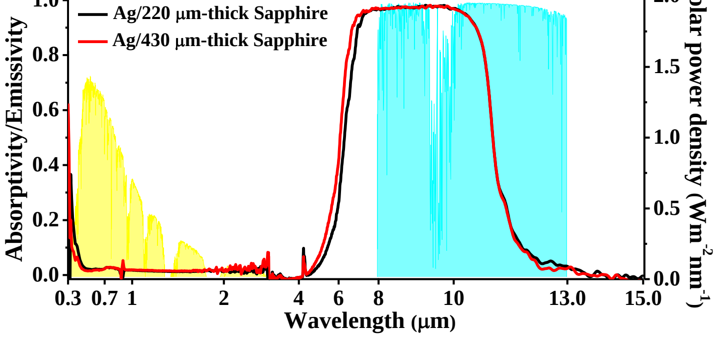

Figure S4. Absorptivity/emissivity spectra of silver-layer-deposited 220-µm- and 430-µm-thick sapphire substrate.

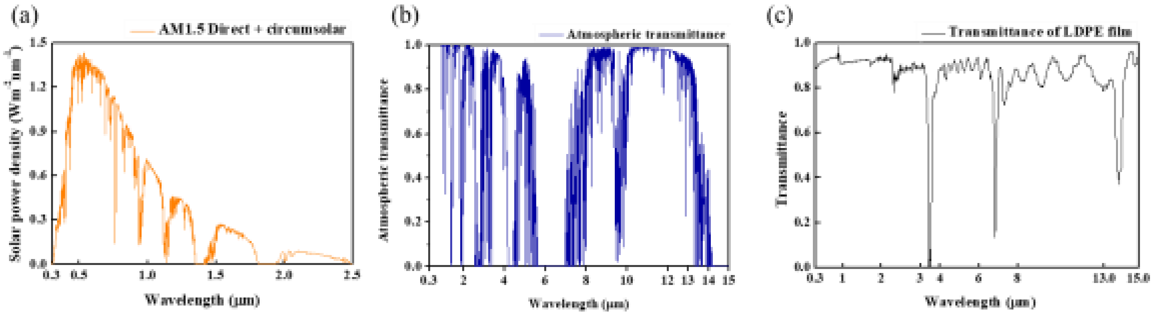

Figure S5. Solar power density with AM 1.5 Direct + circumsolar and atmospheric transmittance acquired from Gemini observatory data.[2] Transmittance in the wavelength range from 0.3 μm to 15 μm of the LDPE film as used in the external cooling temperature measurement.

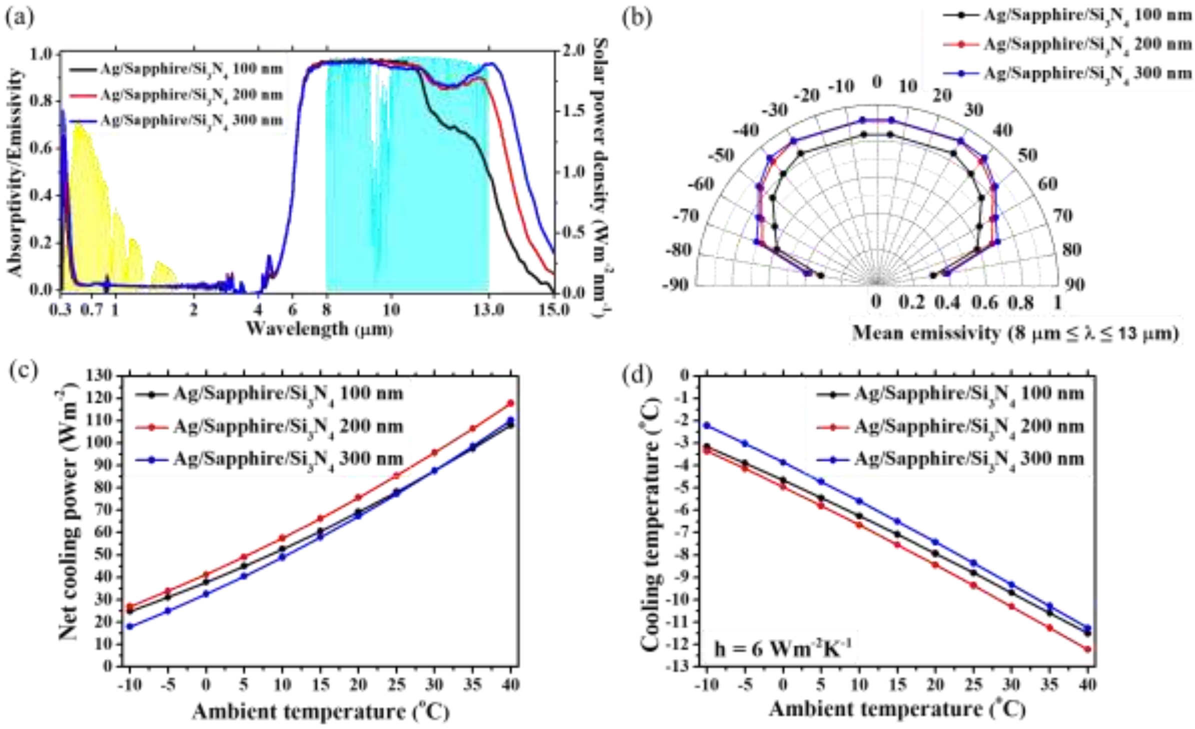

Figure S6. Optimization process of the thickness of the Si_3N_4 thin film for RC 2 (a) absorptivity/emissivity spectra, (b) mean emissivity with variable incident angle, (c) calculated net cooling power, and (d) calculated cooling temperature of RC 2 with Si_3N_4 thicknesses of 100 nm, 200 nm, and 300 nm.

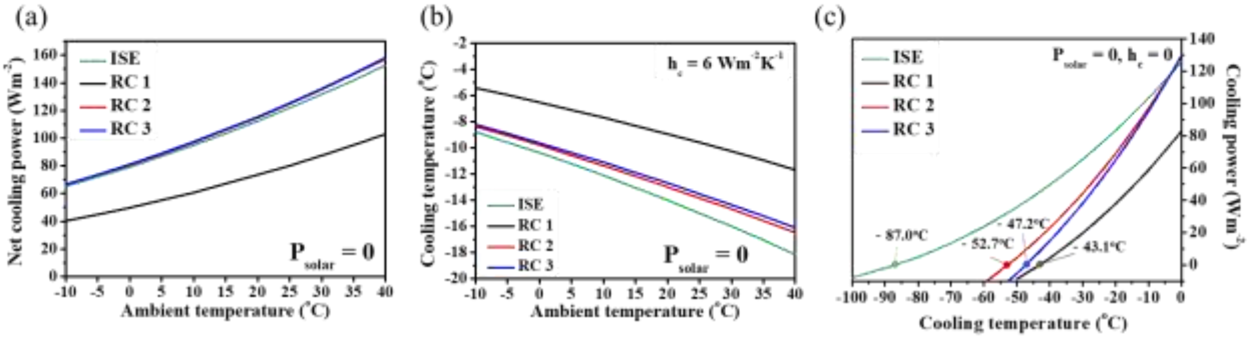

Figure S7. (a) Net cooling power and (b) cooling temperature versus ambient temperature graphs of ISE, RC 1, RC 2, and RC 3 for nighttime. (c) Cooling temperature versus cooling power graph under conditions of zero heat transfer coefficient and ambient temperature of 300 K.

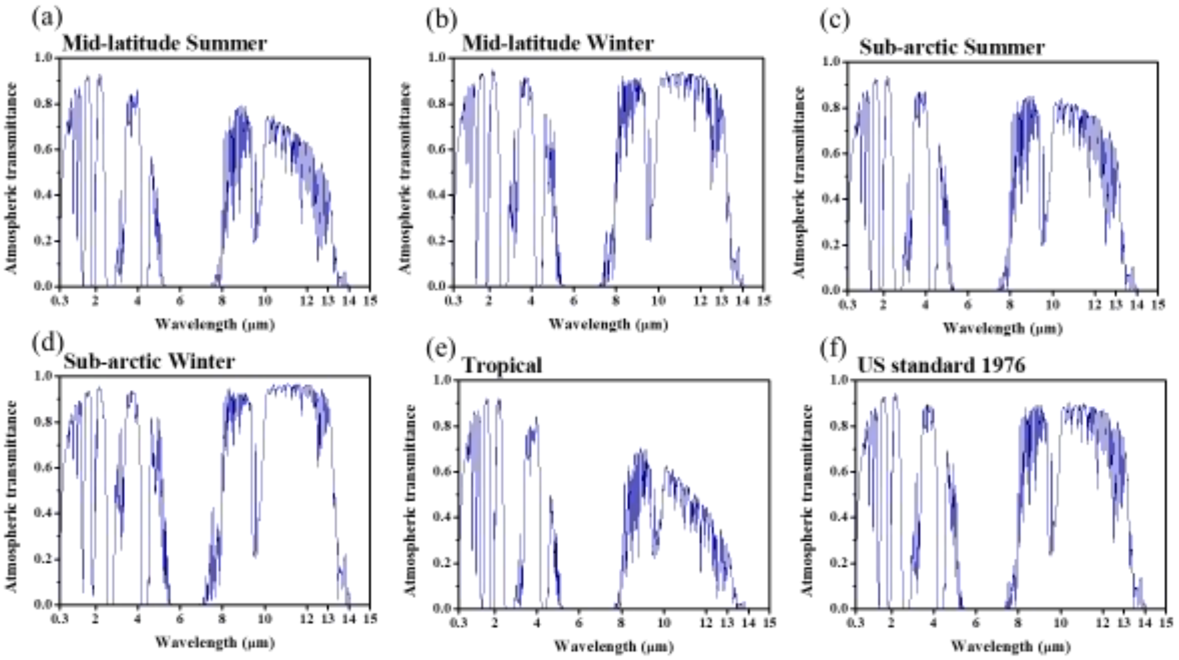

Figure S8. Different atmospheric transmittance values of (a) Mid-latitude Summer, (b) Mid-latitude Winter, (c) Sub-arctic Summer, (d) Sub-arctic Winter, (e) Tropical, (f) US standard 1976 by locations and seasons which are characterized by ground temperature and water vapor column acquired from MODTRAN 6.[3]

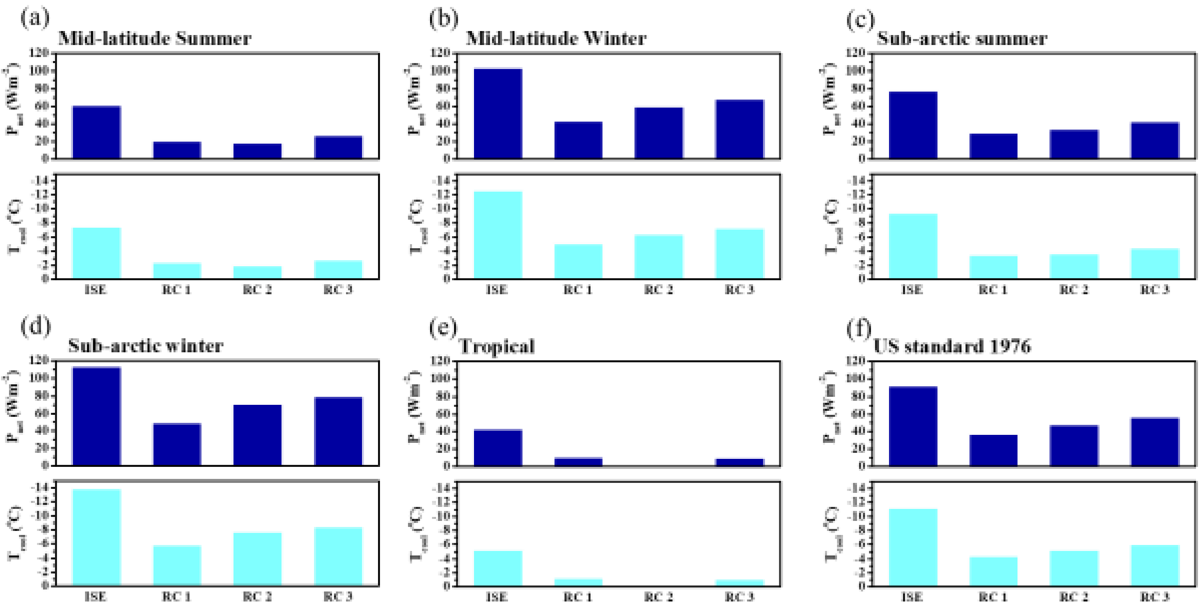

Figure S9. Calculated net cooling power and cooling temperature graphs of ISE, RC 1, RC 2, RC 3 from different atmospheric transmittance models of (a) Mid-latitude Summer, (b) Mid-latitude Winter, (c) Sub-arctic summer, (d) Sub-arctic winter, (e) Tropical, (f) US standard

1976 in Figure S8 with condition of heat transfer coefficient of 6 $Wm^{-2}K^{-1}$ and solar irradiance of 888 Wm^{-2}.

Table S1. Averaged absorptivity in the solar spectral region and emissivity in the atmospheric transparency window of the absorptivity/emissivity spectra of the silver-layer-deposited 220-µm- and 430-µm-thick sapphire substrate.

Sample/optical properties	Ag/220-µm-thick sapphire	Ag/430-µm-thick sapphire
$\alpha_{mean}(0.3-2.5\ \mu m)$	0.026	0.028
$\varepsilon_{mean}(8-13\ \mu m)$	0.615	0.610

Reference

[1] M. Querry, *US Army Chem. Res. Dev. Eng. Cent. (CRDC), Aberdeen Proving Ground, MD* **1985**.

[2] S. D. Lord, *A New Software Tool for Computing Earth's Atmospheric Transmission of Near- and Far-Infrared Radiation*, Vol. 103957, Ames Research Center, Moffett Field, CA, USA **1992**.

[3] A. Berk, P. Conforti, R. Kennett, T. Perkins, F. Hawes, J. Van Den Bosch, in *Work. Hyperspectral Image Signal Process. Evol. Remote Sens.*, **2014**.
